# Disinfectant Performance of a Chlorine Regenerable Antibacterial Microfiber Fabric as a Reusable Wiper

**DOI:** 10.3390/ma12010127

**Published:** 2019-01-02

**Authors:** Cheng Huang, Yongbang Chen, Gang Sun, Kelu Yan

**Affiliations:** 1 College of Chemistry, Chemical Engineering and Biotechnology, Donghua University, Shanghai 201620, China; huangch1988@yeah.net (C.H.); jhsd0204@163.com (Y.C.); 2 National Engineering Research Center for Dyeing and Finishing of Textiles, Donghua University, Shanghai 201620, China; 3 Division of Textiles and Clothing, University of California, Davis, CA 95616, USA

**Keywords:** fabric, N-halamines, antibacterial, environmental health security, food hygiene security

## Abstract

Rechargeable disinfectant performance of a microfiber fabric grafted with a halamine precursor, 3-allyl-5,5-dimethylhydantoin (ADMH), was tested in an actual use situation in a university student dining hall. The precursor was successfully incorporated onto the surfaces of polyester fibers by using a radical graft polymerization process through a commercial finishing facility. The N–H bonds of ADMH moieties on the fibers can be converted to biocidal N–Cl bonds, when the fabrics are washed in a diluted chlorine bleach containing 3000 ppm available chlorine, providing a refreshable disinfectant function. By wiping the surfaces of 30 tables (equivalent to 18 m^2^) with wet chlorinated fabrics, both *Staphylococcus aureus* and *Escherichia coli* in concentrations of 10^5^ CFU/mL were totally killed in a contact time of 3 min. The disinfectant properties of the fabrics were still superior after 10 times successive machine washes (equivalent to fifty household machine washes), and rechargeable after wiping 30 tables before each recharge. Recharging conditions, such as temperature, time, active chlorine concentration and pH value of sodium hypochlorite solution, as well as the addition of a detergent, were studied. The product has the potential to improve public safety against biological contaminations and the transmission of diseases.

## 1. Introduction

Bacterial transmissions in air and their colonization on surfaces contribute significantly to outbreaks of infectious diseases, and are a great threat to public health [1,2,3]. Surface contaminations by microorganisms are a major cause of transmission of infectious diseases [4]. Contact transmissions of *Staphylococcus aureus* (SA) and multidrug-resistant *Staphylococcus aureus* (MRSA) are the major causes of MRSA spread from hospitals to public spaces [5,6]. A common practice to control and reduce disease transmission in healthcare facilities and public spaces is to disinfect contaminated surfaces with a bleach and wipe with a cloth. In recent years, reusable microfiber wipes and mops have been widely adopted by these facilities, which are able to adsorb dusts, particles, and microbes effectively because of their large surface areas [7,8,9,10,11,12]. Meanwhile, chlorine bleach is still an economical disinfectant, commonly used in hospitals and food processing facilities [7]. Thus, biocidal and chlorine bleach rechargeable microfiber cloth could be an ideal reusable disinfecting material for cleaning surfaces of furniture, computer keyboards, and even floors. Most commercially available microfiber wipes do not have any disinfectant ability. Antimicrobial finishing technologies are available for textile treatments, but mostly use heavy metals, quaternary ammonium salts, and phenolic compounds as biocides [13,14,15,16,17,18,19]. These biocides generally need long contact times to kill microorganisms, and cannot disinfect solid surfaces rapidly. More importantly, these antimicrobial chemicals are not rechargeable after uses. With the demand for improved biological protections in public places, environmentally friendly rechargeable and reusable antibacterial wipes should be developed [19,20,21,22].

In recent years, halamine biocidal chemistry has been developed and applied in textiles and polymers to provide chlorine regenerable antibacterial functions [23,24,25,26,27,28]. Various cyclic and acyclic halamine precursors can be grafted onto polymers and the surfaces of textiles [23,24,25,26,27,28]. Halamine structures, in combination with other antibacterial agents, have been grafted onto fabrics to provide unique antimicrobial properties [29]. However, surface chemical modification of chemically inert polyester fibers was not very successful until a controlled radical graft polymerization process, which can incorporate many hydrophilic and hydrophobic vinyl monomers onto polyester surfaces, was developed recently [28,30,31]. 

**Figure 1:**
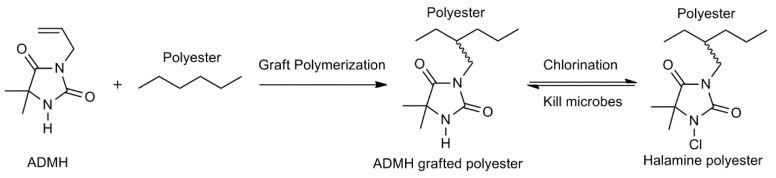
Scheme of grafting 3-allyl-5,5-dimethylhydantoin (ADMH) onto polyester, and chlorination and antibacterial function of the product.

In this study, a precursor of the biocidal halamine chemical, 3-allyl-5,5-dimethylhydantoin (ADMH), was grafted onto surfaces of polyester fabrics by following the controlled radical polymerization reaction, using a commercial finishing facility [28,30,31] (Figure 1). After chlorination of the treated fabrics in a diluted sodium hypochlorite solution, N–H bonds in the grafted ADMH of the fibers were transformed to biocidal N–Cl bonds [32,33]. The N–Cl groups are stable in air and water and provide biocidal functions, but can slowly release free chlorine, depending on the corresponding dissociation constants of their halamine structures [34]. Both combined and free chlorine can instantly kill most common pathogens on contact.

In this paper, the ADMH grafted polyester microfiber cloth was evaluated in actual dining hall settings for the first time as a rechargeable disinfectant wipe. Varied application conditions, such as temperature and concentration of the diluted sodium hypochlorite solution, recharging time, and addition of detergent were investigated. 

## 2. Material and Methods

### 2.1. Chemicals and Materials

Fabrics of polyester/polyamide filament (80% polyester, 20% polyamide, 168D/72F/16P, 250 g/m^2^) were provided by Suzhou Desaisi Commodity Co., Ltd., Suzhou, China. 3-Allyl-5,5-dimethylhydantoin (ADMH), poly(ethylene glycol) diacrylate (PEG-DIA, MW600), and Tergitol XJ were industrial products. Benzoyl peroxide (BPO, Aibi Chemistry Preparation Co. Ltd., Shanghai, China) and ethyl benzoate (EB, Damao Chemical Reagent Factory, Tianjin, China) were analytical reagents. Nutrient agar powder, sodium chloride, and sodium hypochlorite were purchased from Sinopharm Chemical Reagent Co., Ltd. Tryptone (OXOIDTM, Unipath Ltd., Basingstoke, UK) and yeast extract (OXOIDTM, Unipath Ltd., Basingstoke, UK) were biological reagents. Another antibacterial reagent, Reputex 48, was purchased from Arch Biocides Company, Atlanta, GA, USA.

### 2.2. Instrumentations

Attenuated total reflection-Fourier transformed infrared (ATR-FTIR) spectra were obtained using an ATR accessory of a Nicolet 6700 spectrometer (Thermo Fisher Scientific, Waltham, MA, USA). The treated fabrics were directly measured in the range of 4000–400 cm^−1^ at a resolution of 4 cm^−1^. Surface morphology of the grafted and pristine fabrics was observed by a HITACHI/TM-1000 (Shimadzu, Tokyo, Japan) scanning electron microscope (SEM). The samples were plated with gold. Laundry tests were conducted by using a Washtec-P A2 washing machine (Roaches International Ltd., Birstall, West Yorkshire, Egnland) following AATCC test method 61-2A. 

### 2.3. Chemical Finishing Process

The fabrics were modified by a two-dip-two-pad process. The fabrics were padded with 90% pick up after dipping in a finishing solution containing 7% wt ADMH, 2% wt benzoyl peroxide (initiator), 2% wt PEG-DIA, 2% wt Tergitol XJ, and 10% wt ethyl benzoate. The fabrics were dried at a temperature of 80 °C for 1 min. The fabrics were then dipped and padded (about 90% pick up) for a second time. Finally, the fabrics were cured at a temperature of 180 °C for 1.5 min.

Control fabrics were treated using a commercial antimicrobial agent, Reputex 48, according to following procedure. A finishing solution of Reputex 48 was prepared in 2% (on weight of fabric) with a liquid to fabric ratio 20:1 (*w*/*w*). The fabrics were soaked in the solution and treated in a Datacolor AHIBA IR Pro lab dyeing device for 40 min at 130 °C.

### 2.4. Chlorination and Analytical Titration

A piece of the grafted fabric was immerged in a sodium hypochlorite solution containing about 3000 ppm active chlorine for 30 min. The liquid to fabric ratio was 50:1 (*w*/*w*). The fabrics were then rinsed with plenty of water and dried in air. The amount of active chlorine loaded on the fabrics was determined by an iodometric/thiosulfate titration method [23,24]. In this procedure, a precisely measured amount of the chlorinated fibers was added into a 10 mL KI solution (10% wt), 10 mL H_2_SO_4_ solution (10% wt), and 50 mL distilled water. After shaking the container for 30 min, 2 mL of a 10% wt soluble starch solution was added to the mixture. The dark blue mixture was then titrated with 0.001N sodium thiosulfate, until the color of the solution and the fabric disappeared at the end-point. The amount of active chlorine of the bleached grafted fabric was calculated based on the following equation:

Cl (ppm) = (V1 − V2) × N × 35.45 × 100/(2 × W)(1)

where V1 and V2 represent the volumes (mL) of sodium thiosulfate used in titration of the unbleached grafted sample and bleached grafted sample, and W (g) is the weight of the fabrics.

### 2.5. Stability and Durability Testing

American Association of Textile Chemists and Colorists (AATCC) test method 61-2004 was used to test washing durability of the grafted fabrics. In this process, the fabrics were washed with 150 mL 0.15% AATCC standard detergent solution at 49 °C for 45 min, and 50 stainless steel balls were added into the cup. The fabrics endured 10 washing cycles, with each washing cycle equivalent to five household machine washings. The stability and durability of grafted fabrics were judged by measuring the changes of the active chlorine on the fabrics before and after repeated machine washings.

### 2.6. Qualitative Antibacterial Activity

A 0.85 % wt NaCl solution, tweezers, 25 mL glass bottles (each bottle contained 0.1 g cotton ball), and 250 mL glass bottles were sterilized together at 121 °C for 15 min. To ensure complete sterilization, all solution and glassware were radiated under UV light for 30min on an ultra-clean bench. Sterile NaCl solutions of 5 mL and 30 mL were then added into 25 mL and 250 mL bottles, respectively. The antibacterial cleaning properties of the cloth were tested in a university dining hall. Both antibacterial fabrics and common fabrics (as control samples) in the same size (30 cm × 30 cm) were immersed in a diluted chlorine bleach solution (containing 3000 ppm available chlorine) at room temperature for 30 min. Both fabrics were then air dried, after rinsing with certain amounts of tap-water. Finally, the measurement was conducted as follows.

**Figure 2:**
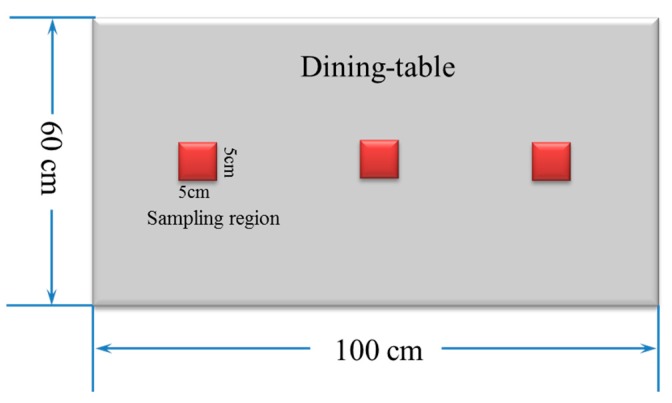
A sketch of a dining table and sampling regions.

First, one sterilized cotton ball was soaked in a 25 mL bottle containing 0.85% wt NaCl to swab the first square in one table for 10 s, and then was placed into the bottle, as shown in Figure 2 (red squares). Another two squares were swabbed by the same method. Thus, three 25 mL bottles represent amount of bacteria on one table surface. Second, a dry, chlorinated microfiber cleaning cloth and a control (unchlorinated) sample were both wetted with 100 mL sterile distilled water. A total of 30 dining tables were wiped successively, following the same procedure as the cotton swabs with the two pieces of fabric. A 3.5 cm × 3.5 cm sample was cut off from each fabric after wiping one, ten, twenty, and thirty tables, and put into a 250 mL bottle containing 30 mL sterilized NaCl solution. After that, all bottles were sealed with caps. The active chlorine remaining on the surface of antibacterial cleaning cloth was also examined after wiping one, ten, twenty, and thirty tables. The third step repeats the first step, except that the cotton balls just swabbed the first, the tenth, the twentieth, and the thirtieth tables, which had already been wiped with the disinfectant cloth or a control sample.

**Figure 3:**
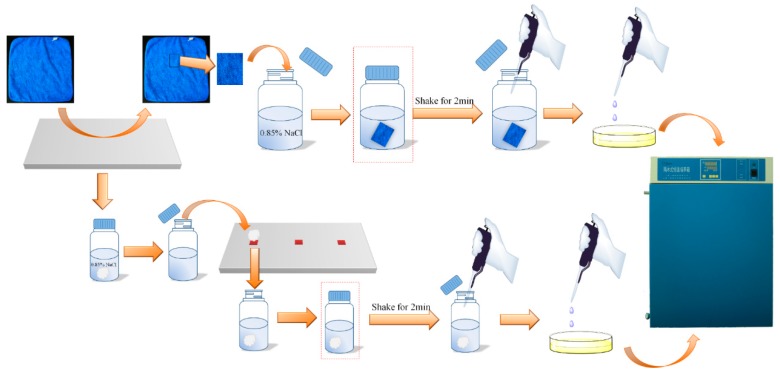
A scheme of the wiping test procedure.

These bottles were shaken vigorously for 2 min. A 0.3 mL volume of the solution was taken out from each 250 mL bottle with a pipette, and dropped onto agar medium. For the 25 mL bottles containing the cotton swabs, a total of 0.3 mL solution was taken out from the three bottles (0.1 mL solution from each bottle). The nutrient agar plates were put into an incubator at 37 °C for 24 h. A scheme of the procedure is shown in Figure 3.

### 2.7. Rechargeability of Fabrics

After wiping 30 tables in the refectory, the antibacterial microfiber wipe was immerged again in the bleach solution containing 3000 ppm active chlorine. The fabric with a wet pick-up of 150% was then dried in the air. The amount of active chlorine and antibacterial property were examined with the methods described in Section 2.4 and Section 2.6, respectively. This process served as a testing cycle and was repeated 5 times.

### 2.8. Quantitative Testing of Antibacterial Activity

A modified American Association of Textile Chemist and Colorists (AATCC) method 100 was adopted to examine the antibacterial properties of the disinfectant samples against *S. aureus* (ATCC 6538) and *E. coli* (ATCC 8099). Samples of 3.5 cm × 3.5 cm were cut off from the fabrics. Two pieces of the control samples were sterilized in the autoclave at 121 °C for 15 min. The control samples and chlorinated samples were then placed in a sterilized container, and 0.6 mL of an aqueous suspension containing about 10^5^ CFU/mL of *S. aureus* or *E. coli* was dropped onto the samples. After different contact times, the samples were placed into 60 mL of 0.05% sodium thiosulfate aqueous solution to quench any active chlorine which might still be present on the fabrics. The solution was then vortexed for 3 min so that the bacteria could be suspended homogeneously in the solution. The suspension was then serially diluted and dropped onto different zones of a nutrient agar plate. The plates were then incubated at 37 °C for 18 h (*S. aureus*) or 10 h (*E. coli*). The antibacterial efficiency was calculated by the following equation.

R(%) = (A − B)/A × 100(2)

where A is the number of bacteria counted from the control fabrics, and B is the number of bacteria counted from the grafted and bleached cloth.

## 3. Results and Discussion

### 3.1. Characterization and Stability

**Figure 4:**
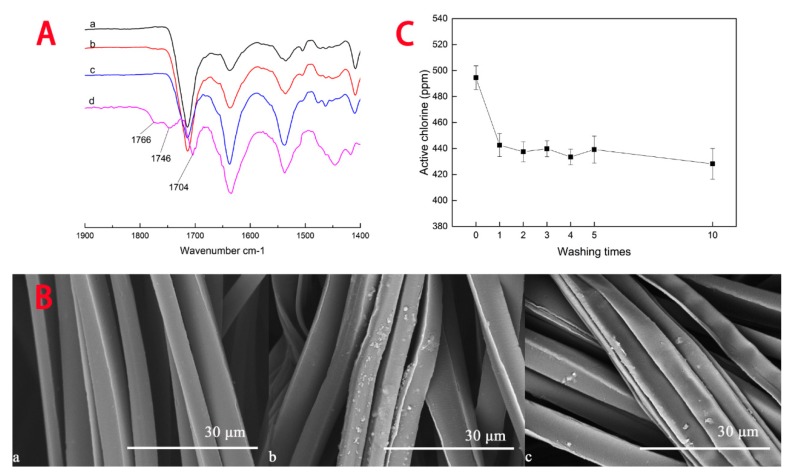
Stability analysis: (**A**) FTIR-ATR spectra of (**a**) pristine fabric, (**b**) grafted fabric, (**c**) ten times washed grafted fabric, and (**d**) difference spectrum, subtracting (a) from (c); (**B**) Scanning electron microscope (SEM) micrographs of (**a**) pristine fabric, (**b**) grafted fabric, (**c**) ten times washed grafted fabric; (**C**) Washing stability of grafted fabrics. Symbol: Mean ± SD.

The fabrics were characterized by FTIR-ATR spectroscopy. Figure 4A shows the FTIR-ATR spectra of pristine fabric (a), grafted fabric (b), ten times washed grafted fabric (c), and subtraction spectrum of line a from line c (d). Because the fabrics are made of PET/nylon filaments, characteristic carbonyl bands of ADMH are similar to the bands of the polymers, but, after subtracting spectrum a from c, some peaks appeared in the resulting spectrum. The peak at 1766 cm^−1^ and 1746 cm^−1^ are attributed to two characteristic carbonyl groups of ADMH hydantoin ring, respectively [26,27]. This is the evidence that the antibacterial precursor has been grafted on the fabrics successfully. SEM images, shown in Figure 4B, provide morphological evidence of the grafted fabrics. Comparing images of (a) with (b) in Figure 4B, it is revealed that the grafted ADMH polymers scattered on the surfaces of the filaments. 

The overall reaction indicates that BPO could initiate the radical graft polymerization reaction of ADMH on the fabrics, confirming the reaction reported in the literature [28,30,31]. After ten washes, the grafted ADMH survived the hard friction and laundry conditions and remained on the surface of the fabric, due to stable covalent bonding. Although ADMH can react with polyester fibers steadily, the grafting reactions occur more efficiently on surfaces of polyamide fibers, since generation of polymeric radicals on weak N–H bonds is easier. Furthermore, grafting ADMH onto defective areas and ends of polyester fibers has been reported using regular graft polymerization process [24]. However, due to the use of the controlled graft polymerization process, the grafting reaction was improved significantly, compared with the literature [24]. Most grafting reactions occurred on the surfaces instead at the ends of the fibers (Figure 4B(b)).

Figure 4C shows the influence of washing times on active chlorine contents of the grafted fabrics. After the first washing, the amount of active chlorine loaded on the fabrics declined about 60 ppm, possibly due to the loss of physically adhered ADMH on the surfaces of the samples. Upon further washes, the active chlorine contents of the grafted fabrics were almost unchanged, evidence of the covalent chemical bonding between ADMH and the polymers. Both FTIR and active chlorine results prove that the hydantoin rings grafted onto the surfaces of polyester fibers could survive repeated laundering, and that the treated fabrics could provide durable and rechargeable antibacterial property.

### 3.2. The Antibacterial Property

#### 3.2.1. Qualitative Antibacterial Evaluation and Stability of Active Chlorine

Qualitative antibacterial evaluations of the chlorinated fabrics were conducted in a university student dining hall according to the procedure designed in Figure 2 and Figure 3. The results are shown in Table 1. First, the existence of bacteria on the surfaces of these dining tables was examined, and the results proved the presence of microbes on all table surfaces. A wipe was used to wipe 30 tables consecutively. After consecutively wiping 10, 20, and 30 tables, bacteria counts on the number 10, 20, and 30 table surfaces and the fabrics were measured, which are presented in Table 1. The surfaces of the #10 and #20 tables and the used wipes after wiping the tables were all completely clean, without any residual microorganisms detected. By counting all cleaned table areas per wipe, one chlorinated wipe cloth can possibly clean about 18 m^2^ without recharging, which is significantly powerful in disinfectant functions. After wiping 30 tables, some live bacteria were spotted on the table and in the wipe.

The untreated fabrics served as controls. Following the same procedure, microorganisms were found on both the surfaces of the wiped tables and in the cloth (Table 1). In fact, more bacteria grew on the used wipe, due to the large surface area and hydrophobicity of the fibers, which can probably trap and retain more live bacteria. This result also explains the importance of adding disinfectant function to microfiber wipes. Although the disinfectant ability of the fabrics declined after wiping 30 tables, the bacteria count from the wipe cloth was still lower than that of the unmodified fabrics (Table 1).

**Figure 5:**
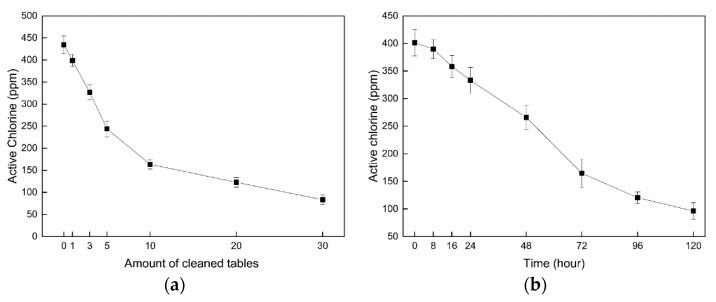
Losses of active chlorine during consecutive cleaning and storage. (**a**) Loss of active chlorine by wiping tables; (**b**) Loss of active chlorine during storage. Symbol: Mean ± SD.

The disinfectant performance of the chlorinated fabrics was superb for the cleaning applications, but relies on the amount of active chlorine on the wipe fabrics. The amounts of active chlorine on the fabrics after repeated wiping of tables and storage were measured to further understand the durability of the biocidal functions. The active chlorine on the fabrics should be consumed continuously during table cleaning, and Figure 5a shows the relationship between the active chlorine loss and the number of cleaned tables. It is obvious that the active chlorine decreased with the increase of tables cleaned. The active chlorine could be consumed by killing bacteria and released to the surfaces of the tables. The loss of the active chlorine was faster when cleaning first 10 tables, and then became slower. Most of the active chlorine loss could be due to water evaporation during the initial cleaning process. Afterward, the loss amount of active chlorine declined relatively. However, since the residual active chlorine on the wipe was still over 100 ppm after 30 uses, the antibacterial properties of the disinfectant power were not affected significantly. 

Storage stability of the wipe cloth was evaluated by measuring active chlorine on the dry fabrics stored under a condition of temperature of 20 °C and humidity of 65%, and the results are shown in Figure 5b. The N–Cl band on the polyester microfiber is not very stable, because of the reactive structures on large surface areas, and easy transformation to an N–H bond after reacting with moisture and microorganisms in atmosphere. After 5 days storage, the active chlorine on the fabrics had dropped conspicuously to below 90 ppm. 

According to the results, we recommend that the chlorinated fabrics should be recharged weekly. The best practice is to bleach the wipe and use it instantly, and to re-bleach it after wiping certain areas. The disinfectant ability of the wipes can be always ensured if they are properly washed in the sodium hypochlorite solution in advance.

#### 3.2.2. Rechargeable Antibacterial Property

**Figure 6:**
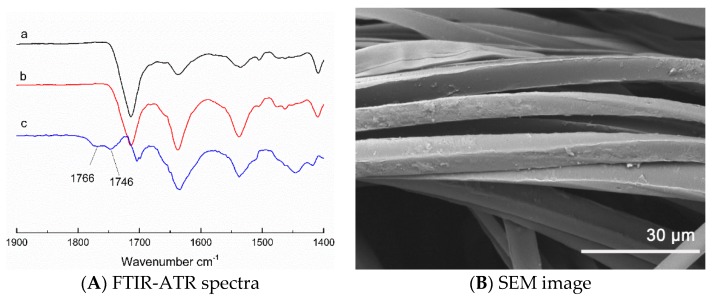
(**A**) FTIR-ATR spectra of the fabric after 5 recharging cycles: (**a**) pristine fabric, (**b**) after cleaning 150 tables, (**c**) difference spectrum of subtracting (a) from (b), (**B**) SEM image of the fabric after 5 recharging cycles.

One of the advantages of using the halamine biocidal wipes is the rechargeable disinfectant properties, which are evaluated with the results shown in Table 2. After wiping 30 tables, the ADMH treated microfiber cloth was immersed in the bleach solution again, and the recharged antibacterial performance of the wipe was as good as that of the freshly made ones. After five recharging cycles, 150 tables have been cleaned, and the characteristic FTIR peaks of hydantoin structure in the grafted microfiber wipes and the SEM images of the fibers were unchanged (Figure 6).

More importantly, the amounts of active chlorine on the recharged samples were almost unchanged, with air dried ones above 400 ppm and the wet samples having above 2000 ppm throughout the five recharges. These active chlorine contents are able to provide powerful, continuous and rapid disinfectant properties. Such results indicate that washing the wipes in diluted bleaching solution will efficiently and effectively recharge the biocidal functions and ensure disinfectant functions on wiped surface areas.

#### 3.2.3. Rapid Antibacterial Function of Fabrics

The antibacterial properties of the grafted fabrics were quantitatively examined against both *S. aureus* and *E. coli* following a modified AATCC test method 100, and the results are shown in Table 3. With contact times increasing from 30 s to 30 min, the fabrics showed improved antimicrobial capabilities (Table 3). The wipe fabrics completely killed of 10^5^ CFU/mL of *S. aureus* after a contact time of 3 min. Furthermore, the fabrics could kill over 90% of *S. aureus* and *E. coli* in a very short 1 min contact time. Such a fast killing rate might be attributable to the high surface area of the microfibers, and certain amounts of free chlorine released from the fabrics.

#### 3.2.4. Comparison with a Commercial Product in Antibacterial Functions

The antibacterial properties of the ADMH grafted fabrics were compared with that of a fabric treated by a commercially available antibacterial agent, Reputex-48, after repeated washing tests. The results are shown in Table 4. The N-halamine microfiber fabrics were chlorinated in sodium hypochlorite solution (containing 3000 ppm available chlorine) after each accelerated washing cycle, before the antibacterial test. Every accelerated washing cycle in this process is equivalent to five household machine washes. The antibacterial properties of the samples were essentially unchanged after 10 washes, revealing excellent rechargeability of the antibacterial functions. On the other side, the fabrics treated with Reputex-48 demonstrated good but reduced antibacterial property with increased washing times. After 10 washes, the antibacterial power of the Reputex-48 finished fabrics was decreased by about 50%, possibly caused by consumption of the active ingredient on the treated fabrics. Obviously, the antibacterial functions on the N-halamine grafted microfiber wipes were more durable because of the rechargeability of the halamine structures.

### 3.3. Factors Affecting Chlorine Recharging Efficiency

The active chlorine on the N-halamine grafted fabric can be repeatedly recharged to provide powerful disinfectant functions. However, recharging efficiency of the active chlorine depends on certain factors of bleaching solution and operation.

**Figure 7:**
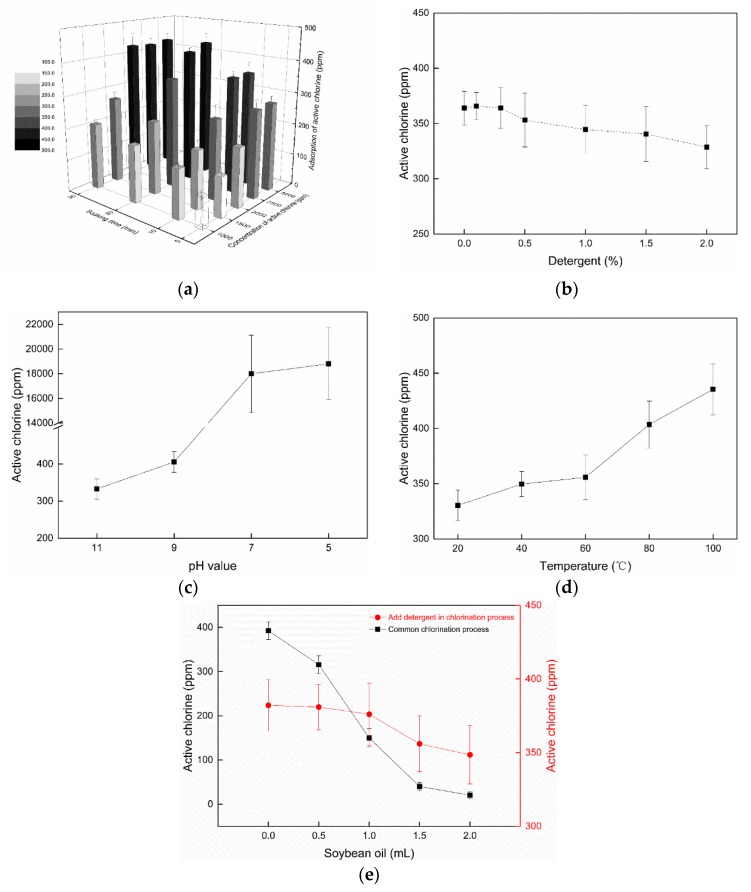
Recharging efficiency affected by different bleaching conditions. (**a**) Soaking time and concentration of bleach solution; (**b**) Addition of a detergent; (**c**) pH of bleach solution; (**d**) Temperature of bleach solution; (**e**) Soybean oil on fabrics. Symbol: Mean ± SD.

The impact of the concentration of the sodium hypochlorite solution and soaking time on the active chlorine content of the wipes is shown in Figure 7a. With varying soaking times and concentrations of the sodium hypochlorite solution, the modified fabrics displayed different amounts of the active chlorine. The active chlorine contents were all below 200 ppm at a low concentration 1000 ppm bleach solution, no matter how long the fabrics were soaked in it. With increasing concentration, the cloth could be charged with more active chlorine in a relatively short time. Overall, the active chlorine charged onto the surface of the fabrics increased with prolonged soaking time and increased concentration of active chlorine.

Due to the hydrophobic nature of polyesters, chlorination of hydantoin rings grafted on the surfaces in aqueous solutions might take more time, especially the under-covered areas ofn the hydrophobic microfiber wipes. However, the loss of active chlorine mostly occurs on the surfaces. Thus, frequent recharging practice still is a better option than soaking in bleach for a longer time, since the lost chlorine can be quickly replenished on the surface areas.

When a wipe is used in cleaning dining tables, it should be able to clean oily stains and dirt on the surfaces, and provide disinfectant functions simultaneously. Thus, surfactants should be added into the diluted bleach solution to enhance the cleaning functions. The addition of some detergents into the bleach solution could influence the recharging efficiency of active chlorine on the wipe fabrics, which was evaluated as well. The results are shown in Figure 7b. When the concentration of a commercial detergent was under 0.5%, the chlorination efficiency of the fabrics was not significantly changed. When the concentration of the detergent was continuously increased from 0.5% wt to 2.0% wt in the bleach solution, a slight decrease of active chlorine amount on the fabrics was noticed. Thus, the bleach solution can contain a low concentration of a surfactant.

The pH value of the bleach solution is another important factor impacting the chlorination process. The amount of active chlorine on the fabrics increased dramatically as the pH value reduced from 11 to 5, as shown in Figure 7c. Under neutral and acidic conditions, the amounts of active chlorine loaded on the fabrics could reach to above 18,000 ppm, a sharp increase versus the ones charged under alkaline conditions. When the pH value was transformed to acidic solution hypochlorite ion concentration, being more favored under alkaline pH, decreased, and concentration of hypochlorous acid (HClO) increased. HClO is a better chlorinating agent of halamine structures. Thus, under acidic conditions, more N–H bonds can be efficiently converted to N–Cl bonds. Moreover, under acidic conditions, the amide group in the polyamide can also be chlorinated, so higher active chlorine contents were detected on the fabrics bleached under pH values of 7 and 5 [31]. 

Regular bleaching and washing of wipes is conducted at room temperature in healthcare and public facilities, but occasionally hot water is used. Therefore, the influence of initial temperature of the bleach solution on chlorination efficiency of the fabrics was investigated, and the results are shown in Figure 7d. High temperature helps the chlorination reaction on the fabric, evidenced by the fact that more than 400 ppm active chlorine was achieved on fabrics in a bleach solution containing 2500 ppm free chlorine for 10 min. A relatively high temperature accelerates chlorine penetration and permeation through the dense hydrophobic microfibers, and makes the N–H bonds on the fabric more easily converted to N–Cl bonds. 

Interestingly, hydrophobic oils could protect the grafted hydantoin from being chlorinated. Different amounts of cooking soybean oil were dropped onto the surfaces of 0.3 g grafted microfiber fabrics before chlorination. The cooking oil attached to the surface of the fabrics affected the chlorination efficiency (Figure 7e). With increasing amounts of the oil dropped on the fabrics, the active chlorine amounts measured on the fabrics decreased dramatically. With 2 mL cooking oil soaking the fabric, the fabric became almost impossible to chlorinate. However, when a detergent (0.5% wt) was added to the dilute bleaching solution, the soybean oil soaked fabric could be chlorinated and the active chlorine amounts loaded on the fabrics were improved, due to removal of the oil by the detergent. 

## 4. Conclusions

An ally hydantoin monomer, ADMH, was successfully grafted onto polyester/polyamide microfiber fabrics using a controlled radical graft polymerization reaction through a commercial finishing facility. FTIR-ATR spectra and SEM images confirmed the successful grafting of the hydantoin structure to the surfaces of the fibers. The ADMH grafted fabrics could be converted to N-halamine biocidal materials in a diluted sodium hypochlorite solution, and more than several hundred ppm of active chlorine were measured on the products. After wiping 30 tables in a university refectory, almost 18 m^2^ of table was disinfected completely by one piece of the fabric without recharging. The wipe fabric could completely inactivate 10^5^ CFU/mL *S. aureus* and *E. coli* in a contact time of 3 min. The disinfectant performance of the wipe cloth was almost unchanged after 10 repeated accelerated washings (equivalent to fifty household machine washings). Comparing with a commercial antimicrobial product, the ADMH grafted fabrics demonstrated superior antibacterial properties and regenerability. Chlorination conditions, including soaking time, temperature, concentration of free chlorine in the bleach, addition of a detergent, and cooking oil attached the fabric, were studied. The ADNH grafted microfiber fabric could be an ideal material to serve as disinfectant wipes for prevention of microorganism transmission.

## Figures and Tables

**Table 1:** The antibacterial qualitative testing results of chlorinated fabrics.

Before Testing	Number of Cleaning Tables ^*^		1	10	20	30
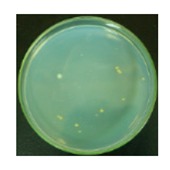	Antibacterial fabrics	Tables	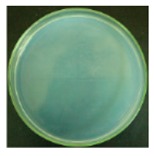	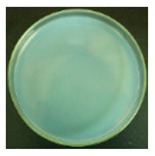	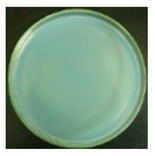	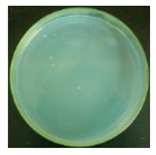
		Fabrics	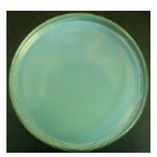	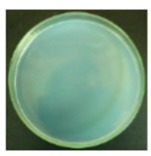	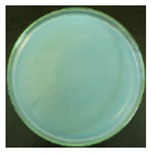	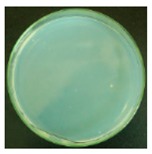
	Untreated fabrics	Tables	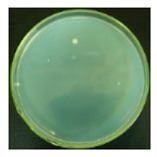	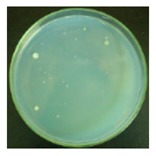	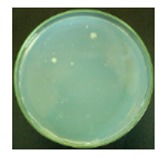	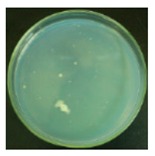
		Fabrics	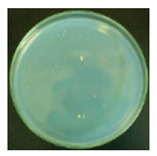	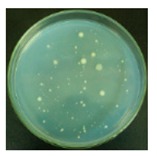	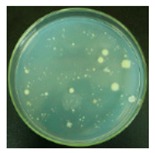	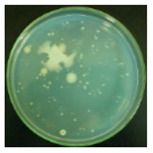

* The area of one table is 60 cm × 100 cm.

**Table 2:** The antibacterial properties and active chlorine contents after repeated chlorine recharges.

Repetition	Antibacterial Property		Active Chlorine (ppm)	
	Tables	Fabrics	Dry	Wet
1	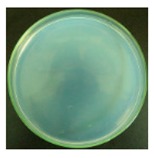	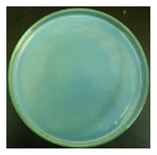	454.3 ± 15.3	2142.1 ± 31.2
2	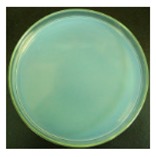	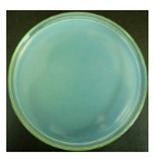	432.2 ± 13.6	2135.3 ± 40.9
3	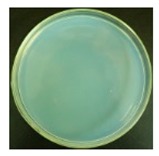	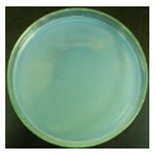	448.7 ± 10.5	2123.9 ± 38.4
4	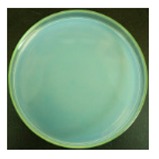	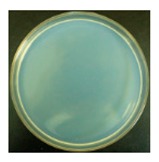	413.6 ± 9.8	2101.2 ± 34.7
5	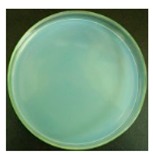	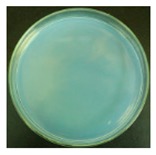	426.6 ± 12.9	2094.7 ± 19.6

**Table 3:** The antibacterial property of fabrics against *S. aureus* and *E. coli*.

Contact Time (min)	Reduction of *S. aureus* ^a^		Reduction of *E. coli* ^b^	
	%	Log	%	Log
0.5	85	0.82	88	0.92
1	95	1.30	94	1.22
3	100	5.40	100	5.34
5	100	5.40	100	5.34
10	100	5.40	100	5.34
30	100	5.40	100	5.34

^a^ Inoculum concentration = 2.51 × 10^5^ CFU/mL; ^b^ Inoculum concentration = 2.19 × 10^5^ CFU/mL.

**Table 4:** Percentage and log reduction of *S. aureus* and *E. coli* after different washing times.

Washing Times	ADMH				Reputex-48			
	*S. aureus* ^a^		*E. coli* ^b^		*S. aureus* ^a^		*E. coli* ^b^	
	%	Log	%	Log	%	Log	%	Log
1	100	5.35	100	5.35	100	5.36	100	5.36
3	100	5.35	100	5.35	100	5.36	100	5.36
5	100	5.35	100	5.35	85	0.82	89	0.96
7	100	5.35	100	5.35	73	0.57	75	0.60
8	100	5.35	100	5.35	70	0.52	69	0.51
9	100	5.35	100	5.35	64	0.44	61	0.41
10	100	5.35	100	5.35	58	0.38	55	0.35

^a^ Inoculum concentration = 2.23 × 10^5^ CFU/mL; ^b^ Inoculum concentration = 2.31 × 10^5^ CFU/mL. Contact time: 1 h.
